# A Novel Sample Processing Method for Rapid Detection of Tuberculosis in the Stool of Pediatric Patients Using the Xpert MTB/RIF Assay

**DOI:** 10.1371/journal.pone.0151980

**Published:** 2016-03-23

**Authors:** Padmapriya P. Banada, Uvistra Naidoo, Srinidhi Deshpande, Farina Karim, JoAnne L. Flynn, Melanie O’Malley, Martin Jones, Oliver Nanassy, Prakash Jeena, David Alland

**Affiliations:** 1 Center for Infectious Diseases, New Jersey Medical School - Rutgers University, Newark, New Jersey, United States of America; 2 KwaZulu-Natal Research Institute for TB/HIV (K-RITH), Durban, South Africa; 3 Department of Microbiology and Molecular Genetics, University of Pittsburgh School of Medicine, Pittsburgh, Pennsylvania, United States of America; 4 Cepheid, 904 Caribbean Drive, Sunnyvale, California, United States of America; 5 Cepheid, Bothell, Washington, United States of America; Emory University, UNITED STATES

## Abstract

**Background:**

Tuberculosis (TB) is difficult to diagnose in children using molecular tests, because children have difficulty providing respiratory samples. Stool could replace sputum for diagnostic TB testing if adequate sample processing techniques were available.

**Methods:**

We developed a rapid method to process large volumes of stool for downstream testing by the Xpert MTB/RIF (Xpert) TB-detection assay. The method was tested and optimized on stool samples spiked with known numbers of *M*. *tuberculosis* colony forming units (CFU), and stools from *M*. *tuberculosis*-infected cynomolgus macaques (*Macaca* fascicularis). Performance was scored on number of positive Xpert tests, the cycle thresholds (Cts) of the Xpert sample-processing control (SPC), and the Cts of the *M*. *tuberculosis*-specific *rpoB* probes. The method was then validated on 20 confirmed TB cases and 20 controls in Durban, South Africa.

**Results:**

The assay’s analytical limit of detection was 1,000 CFU/g of stool. As much as one gram of spiked stool could be tested without showing increased PCR inhibition. In analytical spiking experiments using human stool, 1g samples provided the best sensitivity compared to smaller amounts of sample. However, in Macaques with TB, 0.6g stool samples performed better than either 0.2g or 1.2g samples. Testing the stool of pediatric TB suspects and controls suggested an assay sensitivity of 85% (95% CI 0.6–0.9) and 84% (95% CI 0.6–0.96) for 0.6g and 1.2g stool samples, respectively, and a specificity of 100% (95% CI 0.77–1) and 94% (95% CI 0.7–0.99), respectively.

**Conclusion:**

This novel approach may permit simple and rapid detection of TB using pediatric stool samples.

## Introduction

The World Health Organization (WHO) estimates that over half a million new cases of childhood tuberculosis (TB) occur every year, with an annual mortality of up to 80,000 [1, 2]. Childhood TB accounts for 10–15% of the TB cases worldwide and up to 25% of cases from high TB burden countries [3]. TB can progress very rapidly in children because of their immature immune system [4, 5]. Rapid detection of TB in children should enable more rapid treatment and improved outcomes.

Currently available TB tests perform poorly in children. Most pulmonary TB in adults is diagnosed by smear, culture or molecular tests performed on expectorated sputum [6, 7]. However, children are often unable to expectorate sputum in the volume or quality needed for sputum-based diagnostics. Consequently, sputum smears and cultures for *Mycobacterium tuberculosis* (MTB) are often negative in children with TB [8–10]. Childhood TB can also be diagnosed by testing early morning gastric aspirates [11, 12]. However, this procedure is invasive and the diagnostic yield of gastric aspirates ranges from only 20–40% [8, 12–18]. Children are therefore often treated empirically for TB, based on clinical features, chest X-ray findings, tuberculin skin tests, and contact with an index patient. This approach may lead to both over and under treatment [1]. Furthermore, the lack of bacteriologic culture confirmation makes it impossible to test for drug resistant disease. These factors create an urgent need to develop a rapid and sensitive test that can detect TB and test for drug resistance in children [19].

Stool is a promising sample matrix for pediatric TB tests, because MTB may be swallowed and passed into a child’s stool where it can be easily sampled. MTB is difficult to routinely culture from stool, because other rapidly growing bacteria may overgrow the slowly replicating *M*. *tuberculosis* bacillus. Nucleic acid based amplification tests (NAAT) can detect MTB in stool with good sensitivity [8, 12, 20–22]. The Xpert MTB/RIF (Xpert) assay (Cepheid, Sunnyvale CA), recommended to detect TB in sputum, has also been exploited for extra-pulmonary samples [5, 17, 22, 23]. In previous TB detection studies performed on stool, the Xpert assay detected MTB with a sensitivity of 25–69% and a specificity of 91.7–100% [22, 24–26]. However, the stool processing protocols required centrifugation, which would not be suitable for point of care testing in resource-limited environments. Xpert stool tests also generated unacceptably large numbers of errors (13–25%) [22, 26], probably from sample clogging the Xpert assay cartridge or due to PCR inhibition from stool components. The poor sensitivity of <50% reported in some Xpert stool studies [24, 26] could have been due to retained PCR inhibitors, but may also have been due to the low amounts of sample that were tested (<0.2 g) [24]. Assay sensitivity could be increased if methods were available for testing larger quantities of stool without introducing PCR inhibitors.

Here, we present a novel method to process larger volumes of stool specimens for direct detection of MTB using the Xpert assay. This approach is then validated with pediatric stool samples from TB cases and controls.

## Materials and Methods

### Stool, bacterial strains and culture conditions for analytic laboratory studies

*M*. *tuberculosis* (MTB) strain H37Rv obtained from the American Type Culture Collection (ATCC) (Manassas, VA) was cultured and quantified by plating colony forming units (CFU) on Middlebrook 7H10 agar plates (BD, Franklin Lakes, NJ). *M*. *bovis* BCG Pasteur strain was used as a surrogate for MTB. Pre-quantified frozen MTB and BCG cell stocks were sonicated or vortexed vigorously for 1 to 2 min before making serial dilutions in Middlebrook 7H9 media (BD) in all dilution series. For analytic spiking studies, stools were obtained in the United States from human subjects not suspected of having TB. Stools from cynomolgus (N = 5, *Macaca fascicularis*) and rhesus macaques (N = 4, *Macaca mulatta*), (with active pulmonary TB induced by intrabronchial infection of MTB strain Erdman [27, 28]) or uninfected controls from cynomolgus macaques (N = 5) were freshly collected or stored at 4°C after overnight transport on ice packs.

### Stool processing

**Fig 1:**
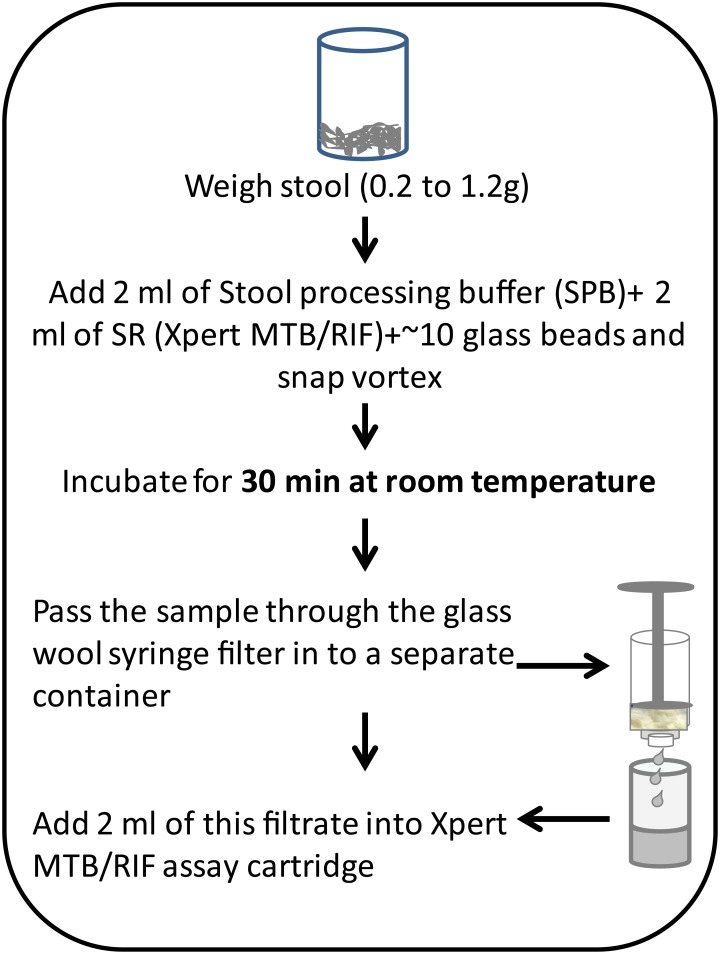
Flow diagram showing how stool was processed in this study.

Between 0.2g to 1.2g of stool, as indicated, were processed following the protocol described in Fig 1. Two mls of a stool processing buffer (SPB), containing AL buffer (Qiagen, Valencia, CA) and 10% Polyvinylpyrrolidone (Sigma Aldrich, St. Louis, MO), two mls of Xpert MTB/RIF sample reagent (SR) (Cepheid), and 3 mm glass beads (Fisher Scientific, Pittsburgh, PA) were added to the mixture. The final stool and buffer combination was mixed by snap vortexing (for <10 seconds), incubated for 30 min at room temperature and then passed through a syringe filter (fitted with glass wool to capture the stool debris) into a clean collection vial (Fig 1). Two ml of this filtrate was then loaded into the sample loading chamber of an Xpert assay cartridge. Subsequent sample processing and PCR were performed in accordance with the manufacturer's recommendations using G3 cartridges in GeneXpert instrument (ver 4.4a).

### Analytical limit of detection (LOD)

One-gram of human TB negative stool was spiked with five different concentrations of *M*. *bovis* BCG (10, 100, 10^3^, 10^4^ and 10^5^ CFU/g) and six different concentrations of MTB strain H37Rv cells (10, 100, 500, 10^3^, 10^4^ and 10^5^ CFU/g). TB negative stool by itself was used as negative control as concentration zero in both experiments. The stool was then treated according to the sample-processing protocol described in Fig 1 for a total of four replicates per CFU concentration. The LOD was defined as the lowest CFU concentration that produced 100% positive results.

### Patient population and study design for clinical validation experiments

Validation studies on fresh stool samples from 20 TB positives and 20 controls were conducted at the KwaZulu-Natal Research Institute for TB and HIV (K-RITH). Children <15 years old were recruited between January 2013 and January 2014 from hospitals and clinics within the Ethekwini region, Durban, KwaZulu-Natal, South Africa. Samples were taken from two groups: TB cases, who were children confirmed to have TB by a positive Xpert MTB/RIF on induced sputum or gastric lavage; and TB-negative controls, who were children that had symptoms or signs suggestive of childhood TB but who had negative Xpert MTB/RIF tests on induced sputum or gastric lavage. Sputum culture was not considered as an enrollment criteria in this study and not all subjects had both Xpert and culture tests performed. TB cases were sourced from case file results obtained through the National Health Laboratory Services (NHLS) GeneXpert laboratories, Durban and reverse traced from these documented results. TB-negative controls were sourced from primary health care clinics or hospitals. Participants were excluded if they had been on more than five days of empiric anti-tuberculosis treatment in the past 6 months, if parental informed consent was unattainable, if child assent for participants 6 to 14 years of age was unattainable, or if they had evidence of extra-pulmonary TB. Participants were withdrawn from the study if there was inadequate stool volume produced. Participants were not followed up after recruitment and sample collection.

Clinical characteristics shown in Table 1 were collected by participant report and chart reviews. TB case definitions used in Table 1 but not in the primary data analysis conformed to national standards as follows: Definite TB disease: At least one of the signs and symptoms suggestive of TB disease and microbiological confirmation of MTB [29, 30]. Probable TB disease: At least one of the signs and symptoms suggestive of TB disease, the chest radiograph being consistent with intra-thoracic TB disease and presence of one of the following: (a) a positive clinical response to TB treatment, (b) documented exposure to a source case with TB disease, or (c) immunological evidence of TB infection [29, 30]. Possible TB disease: At least one of the signs and symptoms suggestive of TB disease and either (a) a clinical response to TB treatment, documented exposure to a source case with TB disease or immunological evidence of TB infection, or (b) Chest radiography consistent with intra-thoracic TB disease [29, 30]. Note, that for study purposes the standard clinical TB case definition did not include the results of molecular tests including Xpert MTB/RIF.

**Table 1:** Baseline clinical findings of recruited TB cases and controls.

	TB Cases^1^ (N = 20)	TB controls^2^ (N = 18)	P value*
Sex (male)	7	10	0.32
Age (<5years old)	8	15	0.009
HIV positive	11	5	0.11
On TB treatment	7	1	0.04
Cough >2weeks	18	5	0.0001
Other constitutional symptoms	20	5	<0.00001
Malnutrition weight-for-height <-2 z-score	7	4	0.48
Positive TB trace contact	14	2	0.0003
Definite TB^3^	4	0	0.1
Probable TB^3^	16	18	0.1
Possible TB^3^	0	0	NA

* 2-tailed Fisher Exact probability values

^1^ TB cases were defined as TB suspects with either a gastric lavage or an induced sputum positive for *M*. *tuberculosis* by the Xpert MTB/RIF assay.

^2^ TB controls were defined as TB suspects with either a gastric lavage or an induced sputum negative for *M*. *tuberculosis* by Xpert MTB/RIF assay.

^3^ Definitions were according to proposed international clinical case definition for intrathoracic *M*. *tuberculosis* disease [29].

### Sample collection

One 5g stool sample was collected from each participant. The timing of bowel motion, sample consistency, relationship to meals, and water intake in the previous 24 hours was noted. The first stool passed in the day was collected for all TB cases and in 16/20 negative controls. Samples were stored at 4°C for maximum one week before testing.

### Laboratory testing

All laboratory testing was performed under a sterile hood in a Biosafety laboratory 3 (BSL3) according to standard operating procedures described in Fig 1.

### Ethics statements

This study was approved by University of Medicine and Dentistry of New Jersey (UMDNJ)-Institutional review board (IRB), which became the Rutgers University IRB upon the merger of UMDNJ and Rutgers on July 1, 2013 (protocols: 2012002020 and 0120110065); by the Biomedical Ethics Research Committee (BREC) of the University of KwaZulu-Natal (protocol: BE233/11) and the KwaZulu Natal Department of Health (protocol: HRKM 072/13). The written consent letter for the participants was also approved by BREC, protocol no: BE233/11 and HRKM 072/13. Participants without written consent were excluded from the study and all the documents are saved on investigators (UN and FK) computers.

The non-human primate stool samples used in this study were obtained from animals who were being investigated for another purpose under IACUC protocol assurance number A3187-01, and specific protocol approval numbers 13122689, 11090030, 1105870, 12060181, and 11110045. The stool samples were obtained only after they had been removed from the animal cages and were slated to be discarded. All experimental manipulations, protocols, and care of the animals for this parent study were approved by the University of Pittsburgh School of Medicine Institutional Animal Care and Use Committee (IACUC). The IACUC adheres to national guidelines established in the Animal Welfare Act (7 U.S.C. Sections 2131–2159) and the Guide for the Care and Use of Laboratory Animals (8th Edition) as mandated by the U.S. Public Health Service Policy. The monkeys were housed at University of Pittsburgh Regional Biocontainment Center (Animal BSL3 facility) and MO collected the stool from monkeys from the cage pan. At the time of collection, none of the monkeys were on treatment. The authors (MJO and JLF) had physical contact with the monkeys.

### Data analysis

Standard statistical analysis (average, standard deviation and t-test) was performed using Microsoft Excel 2000 for Windows. One-way Analysis of variance for independent or correlated samples (ANOVA) was carried out to evaluate the P values using the online ANOVA calculator http://faculty.vassar.edu/lowry/anova1u.html. The reference standard for the diagnosis of TB was the Xpert induced sputum/gastric lavage results. Sensitivity was defined as the percent results with a positive stool result against the GeneXpert induced sputum or gastric aspirate positive cases (+/- 95% exact confidence interval). Specificity was defined as the percent results with a negative result among control patients (+/- 95% exact confidence interval). Two-tailed Fisher exact probability test was used to compare the TB cases to TB controls using the online calculator http://vassarstats.net/odds2x2.html.

## Results

### Optimizing stool quantity

**Fig 2:**
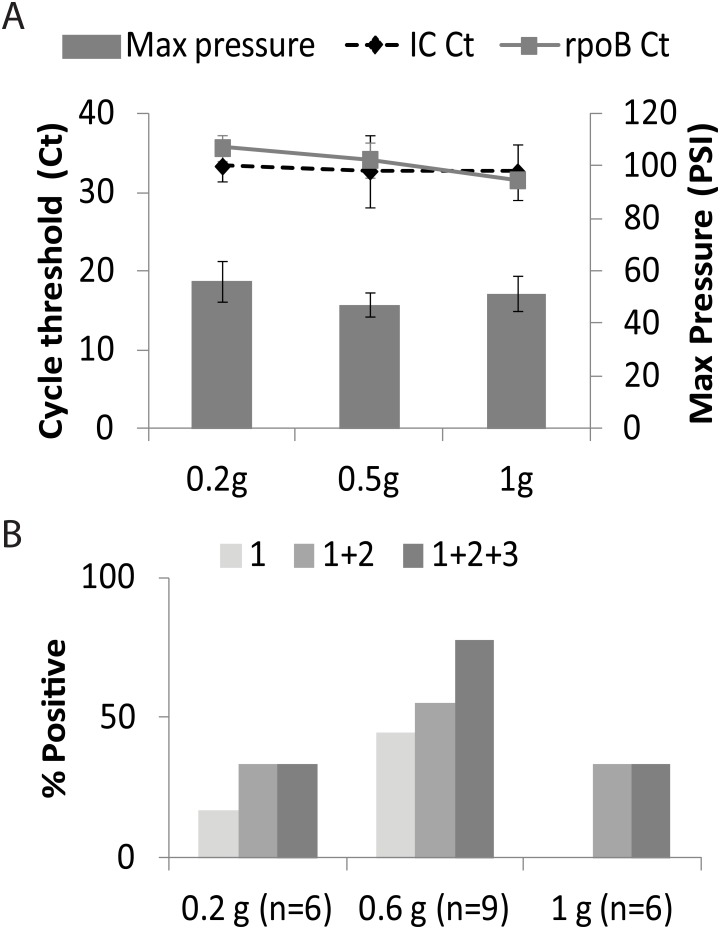
Sample size and sensitivity. **A.** *M*. *bovis* BCG was spiked at 10^3^ CFU/ml into 0.2, 0.5 and 1 g of stool (N = 4). Columns = pressure; lines = Cycle thresholds (Cts). **B.** Stool samples were collected from macaques infected with *M*. *tuberculosis* and three different amounts (0.2g, 0.6g and 1g) were tested. The number in parenthesis indicates number of macaques tested at each volume. The shades of gray on the graph indicate the number of times the test was performed on the same stool sample. Light gray = 1 test only; medium gray = combined data from 2 tests; dark gray = combined data from 3 tests. All the uninfected controls tested (N = 5) were negative by this method.

We studied how much stool sample should be added into our assay to provide the best sensitivity for TB detection [31, 32]. The goal was to introduce the maximum amount of MTB into the assay without degrading PCR performance by introducing excess PCR inhibitors. We spiked 0.2g, 0.5g and 1g of TB negative stool with BCG at final concentrations of 1,000 CFU/g. Samples were processed using the optimized protocol (Fig 1) and tested in the Xpert assay (N = 4 for each stool weight). A delay in the cycle threshold (Ct) of the Xpert assay’s internal control (IC) indicated PCR inhibition [33]. IC Ct values remained at an average 33±3.0 for all stool quantities (Fig 2a), suggesting that our stool processing method effectively eliminates PCR inhibitors in the amounts of stool that we studied. Sample processing pressures measured by the GeneXpert instrument and reported by its normal software were not significantly affected by an increase in stool quantity (P>0.01) and were consistently below 60 PSI or approximately one-half of the permitted pressure limit of the assay (Fig 2a). In contrast, minimum rpoB Cts decreased to 29.8±1.8 with 1g samples compared to 32±4.4 and 35±3.4 for 0.5g and 0.2g samples, respectively (P<0.01 between 0.2g and 1.0g, Fig 2a). These results suggest that adding increasing quantities of processed stool into the Xpert assay improves assay sensitivity for MTB detection.

### Comparing stool volumes from macaques with TB

To simulate human patients with active TB, we performed additional stool quantity ranging studies using stools collected from nine macaques with pulmonary TB and five uninfected controls. Macaque stool samples weighing 0.2g, 0.6g or 1g were tested in triplicate following the protocol in Fig 1, (each test effectively sampled a different portion of the same stool sample) to determine the incremental benefit of testing multiple samples. When a single stool sample was tested for each macaque, 1/6 (17%) of the 0.2g, 4/9 (44%) of the 0.6g, and 0/6 (0%) of the 1.0g stool samples were positive for TB (Fig 2b). When two stool samples were tested for each macaque, 2/6 (33%), 5/9 (56%), and 2/6 (33%) of the 0.2g, 0.6g and 1.0g stool samples, respectively were positive for TB. Finally, when three stool samples were tested for each macaque, 2/6 (33%) of the 0.2g stool samples, 7/9 (78%) of the 0.6g stool samples, and 2/6 (33%) of the 1.0g stool samples were positive for TB. These data suggested that 0.6g was the optimal quantity of stool for our assay, and that repeat testing could further improve assay sensitivity. There was no difference in percent positive rate between the two different macaques (cynomolgus vs rhesus). All the uninfected controls tested (N = 5) were negative by this method. We also observed that macaque stool had many physical differences from the human stool. For example, macaque stool is more pellet in form than the semi-solid consistency observed in human stool.

### Analytical LOD

**Fig 3:**
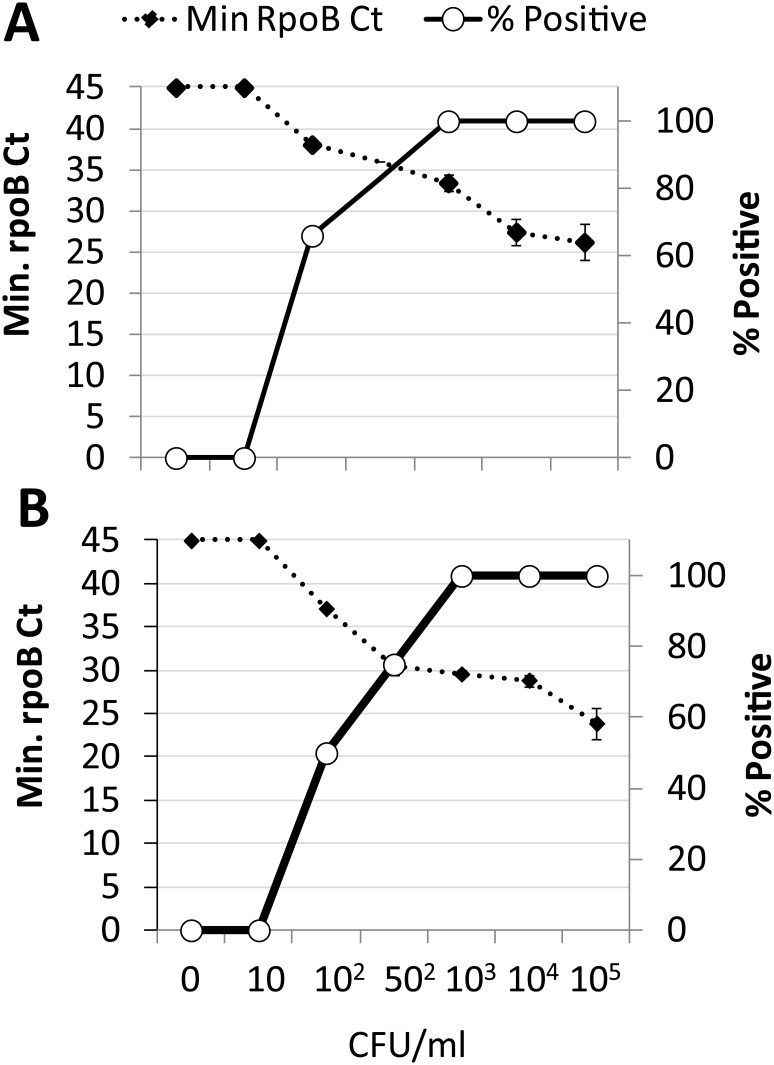
Analytical limit of detection. **A.** BCG and **B.** *M*. *tuberculosis* H37Rv (MTB) was spiked into stool at final concentrations of 10 CFU/g through 10^5^ CFU/g, processed according to our protocol, and tested using the Xpert MTB/RIF assay.

The assay LOD in 1g of stool was evaluated by spiking known numbers MTB CFUs into stool specimens (Fig 3). The assay LOD was 1,000 CFU/g stool for both BCG and MTB. MTB was also detected in 75% and 50% of stool samples spiked with 500 CFU/g and 100 CFU/g, respectively (Fig 3B), whereas BCG was detected 66% of stool samples spiked with 100 CFU/g (Fig 3A).

### Test performance on human clinical samples

**Fig 4:**
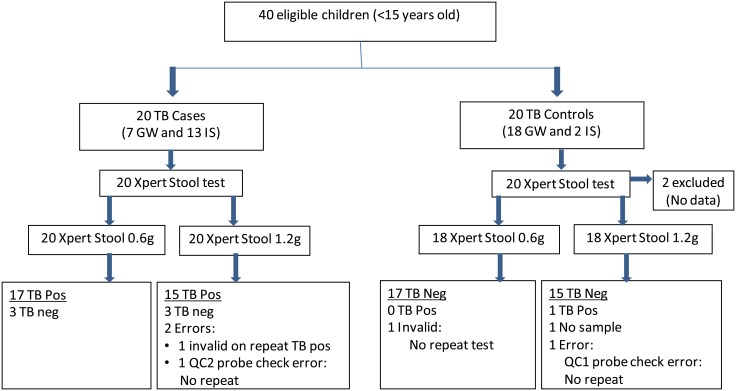
Study flow diagram. Xpert stool testing of 0.6g and 1.2g volume from Xpert induced sputum (IS) and gastric washing (GW) results was performed on above recruited participants. As indicated, repeat testing was performed on a limited stool number of samples that were initially negative.

Twenty TB cases and 20 TB-negative control participants were enrolled in the clinical study. Two controls were excluded due to missing data (Fig 4). Table 1, shows relevant clinical and laboratory characteristics of the participants. Most TB cases and controls fit the definitions proposed by international clinical case definition for intrathoracic *M*. *tuberculosis* [29] of “probable TB”. Sputum MGIT culture results were positive for MTB in only 4 of the 20 TB cases, but cultures were negative in all controls. These 4 cases fit the definition of ‘definite TB’.

Tests performed on 0.6g stool samples were positive in 17/20 TB cases [sensitivity 85% (95% CI 0.6–0.9), Table 2]. Tests performed on 1.2g stool samples were positive in 16/19 stool TB cases [sensitivity of 84% (95% CI 0.6–0.99), Table 2] and 1/20 samples was excluded due to probe check error. The Xpert stool assays performed on 1.2g samples detected TB in 5/6 (83.3%) participants who met our case definition by a positive gastric aspirate, and in 11/13 (84.6%) who met our case definition by a positive induced sputum. Tests performed on 0.6g samples detected TB in 6/7 (85.7%) participants who met our case definition by a positive gastric aspirate, and in 11/13 (84.6%) who met our case definition by a positive induced sputum. The specificity of the assay performed on 0.6g stool samples was 100% [95% CI 0.80–1.00]; and the specificity of the assay performed on 1.2g stool samples was 94% [95% CI 0.67–0.99]. Our assay was positive on tests of both 0.6g and 1.2g stool samples in 4/4 (100%) of participants who were culture positive for MTB in sputum (three participants) or gastric aspirate (one participant).

**Table 2:** Sensitivity and specificity of Xpert stool assay as tested with pediatric clinical samples.

		IS/ GW-Xpert		Total	Sensitivity(95% CI)	Specificity(95% CI)
		Present	Absent			
Xpert-Stool 0.6 g	Present	17	0	17	0.85(0.6–0.9)	1(0.77–1)
	Absent	3	17	20		
Xpert-Stool 1.2 g	Present	16	1	17	0.84 (0.6–0.96)	0.94(0.7–0.99)
	Absent	3	15	18		

In a post hoc subgroup analysis, the stool assay at 0.6g, was positive in 8/11 (72.2%) of HIV positive TB cases, 6/6 (100%) of HIV negative or exposed negative TB cases (HIV children exposed at birth but subsequently tested HIV negative) and 2/2 (100%) TB cases of unknown HIV status. The assay was also positive in 6/7 (85.7%) TB cases who were on TB treatment <5 days, and in 9/13 (69.2%) TB cases who were not on anti-tuberculosis therapy. The four TB cases with positive cultures (3 induced sputum and 1 gastric lavage) were rifampin (RIF) sensitive on phenotypic testing. These four TB cases were also found to have rifampin susceptible MTB in the stool assay. Assay performance did not appear to be affected by stool color and consistency (semi-formed, formed, or liquid), or by water and dietary intake in the 24 hours before stool collection.

## Discussion

We have shown that a simple protocol and buffer set can be used to process stool samples so that they can be tested for MTB with the Xpert MTB/RIF assay. Our method includes two innovative components: a stool processing buffer (SPB), that (along with the Xpert MTB/RIF sample processing reagent—SR) inactivates PCR inhibitors and allows stool to be easily filtered; and a filter configuration that permits easy filtration of the treated stool sample. We found glass wool can be used to filter stool debris more efficiently than the other types of filter materials tested (including cotton, Whatman filters, filter pads, gauze, and glass filter pads). Treating stool samples with SPB and SR and then passing them through our filter as described in Fig 1 resulted in 1 invalid (2.6% of 38 TB cases) from 0.6g and 1 invalid and 2 QC probe check errors (7.8% of 38 TB cases) with 1.2g. Invalids are caused by a delay or an absence of cycle threshold (Ct) from the internal control (IC), which could be due to the introduction of PCR inhibitors from the sample. QC probe check errors are often due to abnormal signals from the assay components within the Xpert MTB/RIF cartridge, which can have multiple causes. In spite of these errors, this performance was significantly better than that achieved by other stool processing methods [21, 22, 24], even though these methods were more complex, tested smaller amounts of stool, and often required additional equipment to permit centrifugation.

It is often difficult to achieve a firm diagnosis of tuberculosis in children [4]. For example, at least two induced sputum samples are required to achieve the sensitivity of a single sputum sample in adult TB suspects [18]. It is likely that pediatric TB is more paucibacillary in the lungs compared to adults and we reasoned that paucibacillary TB would be more easily diagnosed by testing larger volumes of stool. Indeed, in our study of TB infected macaques, the 0.6g stool samples were significantly more positive than the 0.2g stool samples. However, we did not find that the 1.2g stool sample performed better than the 0.6g stool sample in our human participants, even though tests on one 1.2g of stool did suggest that a TB negative control participants had TB. This positive sample in a TB negative participant could represent a false positive diagnosis, or it could represent a true positive in a participant that did not meet our diagnostic criteria for TB. This participant was treated clinically for TB but was not followed up further.

Our study is limited by its small sample size. Our sensitivity and specificity estimates have wide confidence intervals, and an improvement in assay performance with the larger stool size could easily have been missed given the limited number of study participants. Finally, limited facilities made it difficult to confirm by culture the presence of TB in most of our participants who met our case definition of TB positive. Previous studies, however, have shown that the Xpert MTB/RIF assay has a high positive predictive value compared to MTB cultures [34] thus, it is likely that most of our study-defined TB cases truly had TB.

Approximately 10–20% of children from high burden countries develop TB [1, 35, 36]. Molecular diagnostic tools such as the Xpert MTB/RIF have the promise to improve diagnosis of TB in this age group, but obtaining a good quality respiratory sample can be difficult in children. Extra-pulmonary samples such as stool may be easier to obtain and our approach may therefore significantly improve TB detection in children. Furthermore, not all adult TB suspects can produce adequate sputum samples. Therefore, our approach may also be useful in older age groups. Our modified approach to testing stool samples in the Xpert MTB/RIF system is only one aspect of TB diagnosis that must be solved to permit this assay to be easily used in a point of care testing environment. For example, the current GeneXpert test instrument requires a laptop computer and a continuous power source. However, the recently announced introduction of a portable, battery operated, unit that can run Xpert MTB/RIF assays (GeneXpert Omni, http://www.cepheid.com/us/genexpert-omni) may resolve some of the other barriers to point of care testing. In conclusion, the efficient stool sample processing method that we developed appears to simplify TB detection in children. Larger clinical studies are needed to evaluate the full performance parameters and the potential impact of this methodology.
